# Alterations of the Predominant Fecal Microbiota and Disruption of the Gut Mucosal Barrier in Patients with Early-Stage Colorectal Cancer

**DOI:** 10.1155/2020/2948282

**Published:** 2020-03-19

**Authors:** Xia Liu, Yiwen Cheng, Li Shao, Zongxin Ling

**Affiliations:** ^1^Department of Intensive Care Unit, The First Affiliated Hospital, School of Medicine, Zhejiang University, Hangzhou, Zhejiang 310003, China; ^2^Collaborative Innovation Center for Diagnosis and Treatment of Infectious Diseases, State Key Laboratory for Diagnosis and Treatment of Infectious Diseases, National Clinical Research Center for Infectious Diseases, The First Affiliated Hospital, School of Medicine, Zhejiang University, Hangzhou, Zhejiang 310003, China; ^3^Hangzhou Normal University, Hangzhou, Zhejiang 311121, China; ^4^Institute of Translational Medicine, The Affiliated Hospital of Hangzhou Normal University, Hangzhou, Zhejiang 310015, China

## Abstract

Growing evidence indicated that the gut microbiota was the intrinsic and essential component of the cancer microenvironment, which played vital roles in the development and progression of colorectal cancer (CRC). In our present study, we investigated the alterations of fecal abundant microbiota with real-time quantitative PCR and the changes of indicators of gut mucosal barrier from 53 early-stage CRC patients and 45 matched healthy controls. We found that the traditional beneficial bacteria such as *Lactobacillus* and *Bifidobacterium* decreased significantly and the carcinogenic bacteria such as Enterobacteriaceae and *Fusobacterium nucleatum* were significantly increased in CRC patients. We also found gut mucosal barrier dysfunction in CRC patients with increased levels of endotoxin (LPS), D-lactate, and diamine oxidase (DAO). With Pearson's correlation analysis, D-lactate, LPS, and DAO were correlated negatively with *Lactobacillus* and *Bifidobacterium* and positively with Enterobacteriaceae and *F. nucleatum*. Our present study found dysbiosis of the fecal microbiota and dysfunction of the gut mucosal barrier in patients with early-stage CRC, which implicated that fecal abundant bacteria and gut mucosal barrier indicators could be used as targets to monitor the development and progression of CRC in a noninvasive and dynamic manner.

## 1. Introduction

Colorectal cancer (CRC) is one of the leading causes of cancer-related mortality, accounting for the fifth most commonly diagnosed cancer and the fifth most common cause of death by cancer in China [1]. The overall decline or stabilization in the incidence of CRC was noted in several high-income countries [2]; however, the incidence and mortality rates of CRC in China still showed an increasing trend in the past decades, with approximately 376,300 new cases and 191,000 deaths per year [3]. Recently, many previous studies have verified that the human intestinal microbiota, including microbiome, mycobiome, and virome, was one of the important factors associated with CRC [4–10]. Intestinal microbiota, one part of the tumor microenvironment, has attracted increasing attention, as it can affect CRC growth and spread in many ways. A variety of intestinal commensal bacteria and their metabolites are known for triggering inflammatory cascades and oncogenic signaling, thereby promoting genetic and epigenetic alterations in CRC development [11].

Increasing evidence indicated that intestinal dysbiosis plays vital roles in CRC initiation, progression, and metastasis. Our previous study has found that the structures of the intestinal microbiota altered significantly in CRC patients compared to healthy individuals, which found that *Fusobacterium*, *Porphyromonas*, *Peptostreptococcus*, and *Mogibacterium* increased in CRC patients significantly [4]. These bacteria might influence CRC risk via cometabolism or metabolic exchange with the host. Wei et al. demonstrated that *Ruminococcus obeum* and *Allobaculum*-like bacteria were enriched in the feces of 1,2-dimethyl hydrazine treated rats developing precancerous mucosal lesions [12]. Wang et al. also found that an increase of opportunistic pathogens and a reduction of butyrate-producing bacteria may constitute a major structural imbalance of gut microbiota in these CRC patients [13]. Several bacterial species, such as *Fusobacterium nucleatum*, *Peptostreptococcus anaerobius*, and *Bacteroides clarus* [6, 14–16], have been implicated in the development of CRC, which could promote carcinogenesis upon invasion of host cells. In addition, Coker et al. also revealed CRC-associated mycobiome dysbiosis characterized by altered fungal composition and ecology, signifying that the gut mycobiome might also play a role in CRC development [5]. Recently, an international panel of experts from International Cancer Microbiome Consortium delivered a consensus statement on the role of the human microbiome in carcinogenesis [17], which emphasized that future studies should provide direct evidence to demonstrate the roles of the human commensal microbiome in the aetiopathogenesis of cancer including CRC.

Our present study, enrolling confirmed early-stage CRC subjects and matched healthy controls, aimed at assessing the abundant bacteria in the fecal microbiota with quantitative PCR and evaluating the functions of the gut mucosal barrier, which would demonstrate the altered composition of the fecal microbiota in early-stage CRC patients and host response in an easy and rapid way. These results might be helpful for CRC precise diagnosis and personalized treatment targeting on human microbiota.

## 2. Methods

### 2.1. Subjects' Selection

A total of 53 patients who were diagnosed with primary early-stage CRC (aged 46-75 years old) between January 2011 and March 2012 were consecutively recruited from the First Affiliated Hospital, School of Medicine, Zhejiang University, Zhejiang, China. The diagnosis and stages of CRC were based on NCCN clinical practice guidelines in oncology (2010 edition) [18]. None of the patients were on any medications before sample collection. 45 sex-, age-, and body mass index- (BMI-) matched healthy subjects were selected as controls from the same cohorts during a routine physical examination, which were also confirmed by screening colonoscopy and pathology later. The following exclusion criteria were established: obesity (BMI > 30); diabetes; hypertension; family history of CRC; previous colon or rectal surgery; gastrointestinal disorders such as irritable bowel syndrome (IBS) and inflammatory bowel disease (IBD); emergency colonoscopy; known active bacterial, fungal, and viral infections; and the use of probiotics, prebiotics, synbiotics, or antibiotics in the previous month. The study protocol was approved by the Ethics Committee of the First Affiliated Hospital, School of Medicine, Zhejiang University (Zhejiang, China). Written informed consent was obtained from all participants prior to the enrollment.

### 2.2. Sampling

Prior to bowel cleansing for scheduled colonoscopy, fecal samples and blood samples were collected from these participants. We collected approximately 2 g of fresh feces into a sterile plastic cup from these participants and kept these samples in an icebox. Samples for bacterial genomic DNA extraction were transferred immediately to the laboratory and stored at -80°C after preparation within 15 min until use. In addition, blood samples were also collected simultaneously from these participants for intestinal mucosal barrier function analysis. Plasma was also stored at -80°C after preparation within 15 min (Table 1 shows the details of the samples).

### 2.3. Bacterial Genomic DNA Extraction

Fecal genomic DNA was extracted using a QIAamp® DNA Stool Mini Kit (QIAGEN, Hilden, Germany) according to our previous studies [4, 19]. The amount of bacterial genomic DNA was analyzed using a NanoDrop ND-1000 spectrophotometer; the integrity and size of bacterial genomic DNA were checked by electrophoresis. All bacterial genomic DNA was stored at -80°C for further use.

### 2.4. Fecal Abundant Bacteria Analysis

For fecal abundant bacteria analysis, real-time quantitative PCR (qPCR) was performed on ABI Prism 7900HT real-time PCR system (Applied Biosystems, Carlsbad, CA) with a Power SYBR Green PCR Master Mix (Takara, Dalian, China) according to the manufacturer's instructions. The bacterial primer sets and the reaction conditions are shown in Table 2 [19, 20]. SDS 2.4 was used for the data analysis. Triplicate repeats were carried out for all reactions in every analysis with a nontemplate included. The quantity of these bacteria was presented as log10 bacteria per gram of feces (wet weight).

### 2.5. Intestinal Mucosal Barrier Function Analysis

The plasma from each participants was collected for intestinal mucosal barrier function analysis. The parameters of gut mucosal barrier function such as endotoxin (LPS), D-lactate, and diamine oxidase (DAO) were detected by a dry chemical method using the Intestinal Mucosal Barrier Biochemical Index Analysis System (JY-DLT, Beijing Zhongsheng Jinyu Diagnostic Technology Co., Ltd., China) [19, 21, 22]. The experiments were undergone according to the protocols suggested by the manufacturer and conducted within 4 h after plasma extraction.

### 2.6. Statistical Analysis

The quantitative data of fecal abundant bacteria and other parameters in our study are presented as the mean ± standard deviation (SD), the differences between the two groups were evaluated by Student's *t* test, and the correlations between variables were tested by Pearson correlation using SPSS version 20.0 (SPSS Inc., Chicago, IL.), respectively. *p* < 0.05 was considered statistically significant for all analyses.

## 3. Results

### 3.1. Characteristics of the Early-Stage CRC Patients

In our present study, these participants were all newly diagnosed early-stage CRC patients in our hospital, who were not treated with antibiotics, prebiotics, probiotics, and synbiotics in the previous month. Before sampling, these participants have been performed a complete physical examination such as colonoscopy and pathology, serological markers detection, and radiological examination. Most of the CRC patients (50/53) were found to be positive in fecal occult blood testing, while several patients (8/53) occurred severe diarrhoea. In our study, the BMI values and the serum liver function, kidney function, lipid levels, blood sugar, and blood pressure in the CRC patients were not different from healthy controls significantly (*p* > 0.05). However, the serological marker such as carcinoembryonic antigen (CEA) was increased significantly in CRC patients when compared with healthy controls (*p* < 0.05). Our study demonstrated that no any CRC patients were found to be positive in tumor metastasis.

### 3.2. Abnormal Fecal Microbiota in CRC Patients

**Figure 1:**
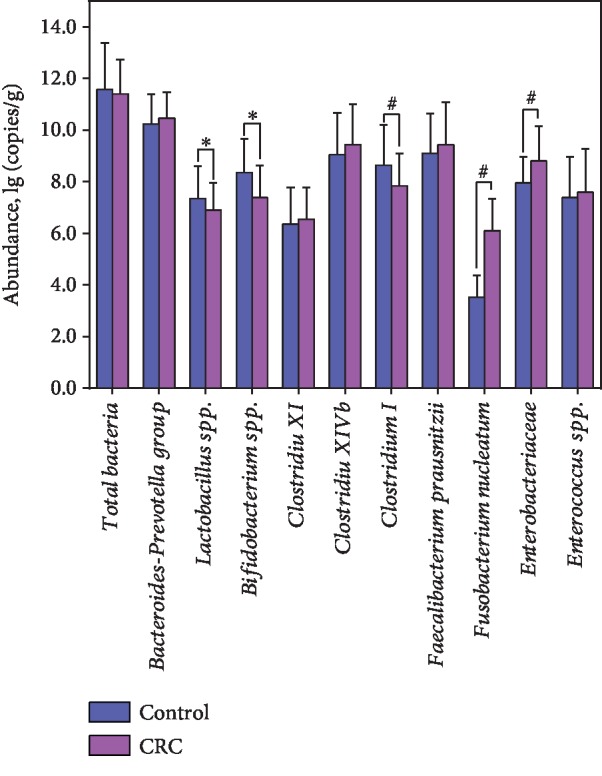
Quantitative real-time PCR analysis of the fecal abundant bacteria in patients with colorectal cancer (log10 copies per gram of fresh feces). ^∗^*p* < 0.05; ^#^*p* < 0.01.

In our present study, we investigated the abundant bacteria in the fecal microbiota by real-time quantitative PCR (Figure 1). The abundant bacteria represented the higher relative abundance of bacteria in the fecal microbiota, which would determine the overall structure and composition of the fecal microbiota. Ten abundant bacteria, which accounted for more than 90% of the total bacteria, were selected to explore the alterations of fecal microbiota in these CRC patients. We found that the copy numbers of the total bacteria were not significantly different between healthy controls and CRC patients. However, the traditional beneficial bacteria such as *Lactobacillus* and *Bifidobacterium* decreased significantly in CRC patients. In addition, the gut-indigenous *Clostridium* included several different clusters, including abundant clusters such as *Clostridium* cluster I, cluster XI, and cluster XIVb. Our data indicated that *Clostridium* cluster I was significantly decreased in CRC patients when compared with healthy controls, while *Clostridium* cluster XI and cluster XIVb were not significantly different between healthy controls and CRC patients. Another bacterium, Enterobacteriaceae, was considered as gut harmful bacteria, which could produce lipopolysaccharide into peripheral blood. We observed that Enterobacteriaceae was significantly increased in CRC patients when compared with healthy controls. *F. nucleatum* was the confirmed carcinogenic bacterium that associated with CRC development. Consistent with the previous studies, our results demonstrated that *F. nucleatum* was significantly increased in CRC patients when compared with healthy controls. Our data indicated that dysbiosis of fecal microbiota was found in CRC patients, which might be contributed to CRC initiation, progression, and metastasis.

### 3.3. Changed Intestinal Mucosal Barrier Function

**Figure 2:**
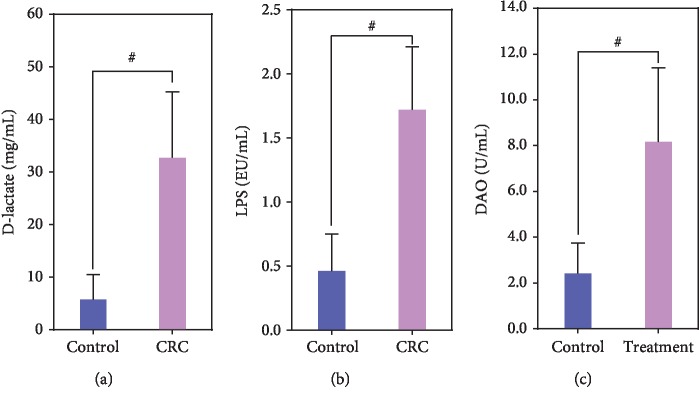
The alterations of the indicators of the gut mucosal barrier in patients with colorectal cancer. (a) D-lactate; (b) LPS; (c) DAO. ^#^*p* < 0.01.

The three parameters such as LPS, D-lactate, and DAO could be used to evaluate the integrity of the gut mucosal barrier. The increased permeability of the gut mucosal barrier was associated with increased systemic and local inflammation, which was closely correlated with the initiation and development of CRC. In our present study, we found obviously disturbed gut mucosal barrier in CRC patients when compared with healthy controls. Our data indicated that plasma D-lactate, a byproduct of bacterial metabolism, increased significantly in CRC patients when compared with healthy controls (*p* < 0.01; Figure 2(a)). LPS, a structural component of the cell wall of Gram-negative bacteria, was a potent inducer of proinflammatory cytokines that activate a systemic inflammatory response syndrome. Elevated LPS level has been reported to be an early marker of impaired mucosal barrier function. In our study, we also observed that the levels of LPS were significantly higher in the CRC patients than that in the healthy controls (*p* < 0.01; Figure 2(b)). In addition, DAO, an enzyme which deaminates histamine and polyamines, has its highest activity in the intestinal mucosa in most mammalian species, including humans. DAO activity has been shown to reflect changes associated with the gut mucosal barrier. Our data indicated that significant differences of DAO levels were observed between CRC patients and healthy controls (*p* < 0.01; Figure 2(c)). Taken together, our results showed that the loss of gut mucosal barrier integrity and function was found in CRC patients, which indicated that the impairment of gut mucosal barrier function might be one of the important factors for the initiation and development of CRC.

### 3.4. Correlations between Differential Bacteria and Indicators of Gut Mucosal Barrier

In our present study, Pearson correlation was used to examine the relationship between differential fecal bacteria and indicators of the gut mucosal barrier (Table 3). We found that the differential fecal bacteria such as *Lactobacillus*, *Bifidobacterium*, and *Clostridium* cluster I correlated with D-Lactate and endotoxin negatively (*p* < 0.05), while *Lactobacillus*, but not *Bifidobacterium* and *Clostridium* cluster I, correlated with DAO negatively (*p* < 0.05). In addition, we also found that *Fusobacterium nucleatum* and Enterobacteriaceae correlated positively with D-Lactate, DAO, and endotoxin (*p* < 0.05). Among these indicators of gut mucosal barrier, strong correlations (Pearson's *r* ≥ 0.50) were observed between *Bifidobacterium* and endotoxin (*r* = −0.752, *p* < 0.000), between *Clostridium* cluster I and endotoxin (*r* = −0.630, *p* < 0.000), between *Fusobacterium nucleatum* and D-Lactate (*r* = 0.809, *p* < 0.000), and between Enterobacteriaceae and endotoxin (*r* = 0.802, *p* < 0.000).

## 4. Discussion

The development of human CRC is influenced by various risk factors, such as diets, genetic, epigenetic, environmental, and metabolic factors, while environmental factors are the predominant trigger for CRC [23]. A growing body of evidence has indicated that human gut microbiota may be an important environmental factor that promotes CRC development [15, 24]. With the advent of high-throughput sequencing platforms “omics” technologies and advanced bioinformatics approaches, the changes of the composition and function of the gut microbiota have been identified in the last decades, and the suspected causative roles and molecular mechanisms of gut microbiota in CRC development have been established by many previous studies. Recently, several bacterial species such as *Fusobacterium nucleatum* and *Peptostreptococcus anaerobius* have been confirmed in the CRC development and progression [14, 25]. These fecal bacteria have been considered as one of the most important elements of the tumor microenvironment. Now, fecal candidate bacteria can be used as novel biomarkers with existing methods, such as real-time qPCR and fecal immunochemical test, for noninvasive diagnosis of CRC [7].

With real-time qPCR, we found that the abundant fecal bacteria changed significantly in these early-stage CRC patients, which indicated that the dysbiosis of fecal microbiota might participate in CRC development and progression. In our present study, ten abundant bacteria were investigated to illustrate the alterations of fecal microbiota in CRC patients. In our present study, the two traditional beneficial bacteria such as *Lactobacillus* and *Bifidobacterium* (belonged to the lactic acid bacteria category) decreased significantly in these CRC patients, which were consistent with previous study [26]. Mendes et al. has found that the abundance of the genera *Lactobacillus* and *Bifidobacterium* increased significantly after microbiota modification by probiotic supplementation [27]. Increasing reports in recent years have shown that these bacteria have demonstrated a host of properties in preventing CRC development by inhibiting initiation or progression through multiple pathways, such as apoptosis, antioxidant DNA damages, immune responses, and epigenetics [28–30]. Although *Lactobacillus* and *Bifidobacterium* may only act before carcinogenesis and have little inhibitory impact on established cancer, these two beneficial bacteria were the most frequently used as probiotics to prevent colon carcinogenesis in animal models or patients, which might help regulate the CRC-related tumor microenvironment. Different from *Lactobacillus* and *Bifidobacterium*, Enterobacteriaceae was often considered as harmful bacteria in the fecal microbiota, which could induce inflammation in the host gut epithelium [31]. As inflammation has been a well-documented risk factor for various form of cancer [32], it was implicated that Enterobacteriaceae participated actively in the development and progression of CRC. Previous studies showed that the actions of Enterobacteriaceae are similar to the prolonged inflammatory response induced by enterotoxigenic *Bacteroides fragilis* [33, 34]. Sun et al. has showed significant enrichment of Enterobacteriaceae in the DMH-induced CRC animal model [35]. They also found that Enterobacteriaceae showed higher relative abundance in the early stages of tumor formation, while it was gradually replaced with other bacteria such as Rikenellaceae, Lachnospiraceae, Ruminococcaceae, and Streptococcaceae [35]. Another potential cancer-promoting bacterium, *F. nucleatum*, was associated with CRC. Previous studies found that *F. nucleatum* is enriched in both the feces and colonic mucosa of CRC patients [4, 36, 37] and plays important roles in colorectal carcinogenesis [38, 39]. *F. nucleatum* may promote colorectal tumor growth and inhibit T cell-mediated immune responses against colorectal tumors [40]. Rubinstein et al. also found that *F. nucleatum* binds E-cadherin on epithelial cells and activates *β*-catenin signaling, driving epithelial cell proliferation [39]. With quantitative PCR and fecal immunochemical test, Liang and their colleagues found that *F. nucleatum* alone can discriminate CRC from controls with a sensitivity of 77.7%, and specificity of 79.5%, which can serve as a novel noninvasive diagnostic method for patients with CRC [7]. Mima et al. also found that the amount of *F. nucleatum* in CRC tissue is associated with shorter survival and may potentially serve as a prognostic biomarker [40]. Bullman et al. observed that treatment of mice bearing a CRC xenograft with the antibiotic metronidazole reduced *F. nucleatum* load, cancer cell proliferation, and overall tumor growth, which indicated that microbiota modulation as a potential treatment for *Fusobacterium*-associated colorectal carcinomas [41]. In addition, we also found that *Clostridium* cluster I decreased significantly in CRC patients when compared with healthy controls. Kostic et al. found that CRC tissues have decreased microbial diversity, including a reduction of certain bacterial genera like *Clostridium* and *Bacteroides* [42]. However, the genus *Clostridium* includes a diverse group of Gram-positive, spore-forming anaerobes [43]. In general, clostridial fermentative metabolism functions by the conversion of hexose sugars to butyrate, acetate, and CO_2_ [44]. Our study firstly found that *Clostridium* cluster I was negatively correlated with CRC development. The present study indicated that the abundant bacteria in fecal microbiota participated actively in the development and progression of CRC.

The gut mucosal barrier is a functional unit organized as a multilayer system, and its multiple functions are crucial for maintaining gut homeostasis. The inherent property of the gut to act as a semipermeable barrier is crucial for the maintenance of health. Dysfunction of the gut mucosal barrier leads to increased translocation of commensal bacteria and their metabolites locally and systemically, triggering the inflammatory response. Gut inflammation results in excess production of proinflammatory cytokines that can in turn increase mucosal permeability by altering intercellular tight junction structure and induce apoptosis of intestinal epithelial cells. Numerous scientific evidences showed a significant association between impaired gut mucosal barrier and gastrointestinal/extraintestinal diseases [45–48]. Previous study has found that progression of colorectal neoplasia has been linked to alterations of tumor microenvironment and gut mucosal barrier function, which facilitate the interaction of microbial products with host pathways [11]. D-lactate, LPS, and DAO were three accepted and convenient indicators to evaluate the integrity of the gut mucosal barrier. The increased permeability of the gut mucosal barrier would lead to bacterial translocation that was accompanied by increasing levels of D-lactate, LPS, and DAO, which finally contribute to a number of intestinal diseases such as CRC. D-lactate, a bydroxycarboxylic acid produced by bacterial fermentation, is a useful indicator of increased gut permeability and gut barrier dysfunction. Our present study found that the level of D-lactate was increased significantly in CRC patients, which was correlated with *Lactobacillus*, *Bifidobacterium*, and *Clostridium* cluster I negatively and with *F. nucleatum* and Enterobacteriaceae positively, confirming the existence of gut mucosal barrier dysfunction. LPS is large molecules found in the outer membrane of Gram-negative bacteria. The increase in LPS is associated with bacterial translocation due to the impairment of intestinal epithelial cell [49]. Previous study has found that gut microbiota dysbiosis alters the intestinal barrier function, increases plasma LPS levels, which promotes endotoxemia, and contributes to the onset and development of CRC [50]. Our study also found that the concentration of LPS increased significantly in CRC patients, which was correlated with *Lactobacillus*, *Bifidobacterium*, and *Clostridium* cluster I negatively and with *F. nucleatum* and Enterobacteriaceae positively. The results suggest that there was an increased intestinal permeability in these CRC patients. DAO is an enzyme mainly produced in the small intestine involved in the histamine metabolism [51]. Yee et al. has found that an established first-line treatment for patients in T2DM, metformin, inhibits DAO activity [52]. Previous study also found that serum DAO activity decreased step-by-step significantly during anticancer drug therapy in human, which may be to serve as a useful predictor of gastrointestinal toxicity due to anticancer drug [53]. Our present data found that the levels of DAO also increased significantly in CRC patients, which was correlated with *Lactobacillus*, *Bifidobacterium*, and *Clostridium* cluster I negatively and with *F. nucleatum* and Enterobacteriaceae positively. Taken together, the low levels of D-lactate, LPS, and DAO in normal conditions increased significantly in CRC patients, suggesting a breakdown of the gut mucosal barrier.

In summary, dysbiosis of the fecal microbiota, which was featured by altered abundant fecal bacteria, and dysfunction of the gut mucosal barrier, which was characterized by increased levels of D-lactate, LPS, and DAO, occurred in patients with early-stage CRC. The abundant bacteria in the feces and the indicators of the gut mucosal barrier in the plasma could be used as targets to monitor the development and progression of CRC in the future.

## Figures and Tables

**Table 1:** Summary information of the samples.

	CRC	Control
Sample size	53	45
Age (years): mean ± SD	52.4 ± 18.8	53.7 ± 16.7
Gender (male/female)	33/20	25/20
BMI	24.8 ± 3.58	23.7 ± 3.67

**Table 2:** Bacterial-specific primer sets for detection of fecal microbiota by qPCR.

PCR specificity	Primer	Sequence (5′→3′)	Annealing temperature (°C)	Amplicon size (bp)
Total bacteria	Uni331F	TCCTACGGGAGGCAGCAGT	58	466
	Uni797R	GGACTACCAGGGTATCTATCCTGTT		
*Bacteroides*-*Prevotella* group	Bac303F	GAAGGTCCCCCACATTG	56	418
	Bac708R	CAATCGGAGTTCTTCG		
*Lactobacillus* spp.	Lac-F	AGCAGTAGGGAATCTTCCA	58	341
	Lac-R	CACCGCTACACATGGAG		
*Bifidobacterium* spp.	Bifid-F	CTCCTGGAAACGGGTGG	55	550
	Bifid-R	GGTGTTCTTCCCGATATCTACA		
*Clostridium* cluster I	CG1-F	TACCHRAGGAGGAAGCCAC	63	700
	CG1-R	GTTCTTCCTAATCTCTACGCAT		
*Clostridium* cluster XI	CG2-F	ACGCTACTTGAGGAGGA	58	141
	CG2-R	GAGCCGTAGCCTTTCACT		
*Clostridium* cluster XIVab	CG3-F	GAWGAAGTATYTCGGTATGT	52	152
	CG3-R	CTACGCWCCCTTTACAC		
*Faecalibacterium prausnitzii*	Fpra-F	GATGGCCTCGCGTCCGATTAG	58	199
	Fpra-R	CCGAAGACCTTCTTCCTCC		
*Fusobacterium nucleatum*	Fn-F	CAACCATTACTTTAACTCTACCATGTTCA	60	112
	Fn-R	GTTGACTTTACAGAAGGAGATTATGTAAAAATC		
*Enterobacteriaceae*	Eco-F	CATTGACGTTACCCGCGAGAAGAAGC	63	195
	Eco-R	CTCTACGAGCTCAAGCTTGC		
*Enterococcus* spp.	ENco-F	CCCTTATTGTTAGTTGCCATCATT	61	144
	ENco-R	ACTCGTTGTACTTCCCATTGT		

**Table 3:** Pearson's correlation coefficients between differential abundant bacteria and indicators of gut mucosal barrier.

	D-lactate (mg/ml)		DAO (U/ml)		LPS (EU/ml)	
	*r*	*p*	*r*	*p*	*r*	*p*
*Lactobacillus*	-0.314	0.043	-0.411	0.006	-0.456	0.002
*Bifidobacterium*	-0.385	0.011	-0.149	0.341	-0.752	0.000
*Clostridium* cluster I	-0.332	0.030	-0.252	0.103	-0.630	0.000
*Fusobacterium nucleatum*	0.809	0.000	0.339	0.026	0.456	0.002
Enterobacteriaceae	0.402	0.007	0.342	0.025	0.802	0.000

## Data Availability

The data used to support the findings of this study are available from the corresponding author upon request.
